# Zinc status is associated with inflammation, oxidative stress, lipid, and glucose metabolism

**DOI:** 10.1007/s12576-017-0571-7

**Published:** 2017-09-30

**Authors:** J. Olechnowicz, A. Tinkov, A. Skalny, Joanna Suliburska

**Affiliations:** 1 Poznan University of Life Sciences, ul. Wojska Polskiego 31, 62-624 Poznan , Poland; 2 Orenburg State Medical University, Sovetskaya St., 6, Orenburg, 460000 Russia; 3 Orenburg State University, Pobedy Avenue, 13, Orenburg, 460018 Russia; 4 RUDN University, Miklukho-Maklay St., 10/2, Moscow, 117198 Russia; 5 Yaroslavl State University, Sovetskaya St., 14, Yaroslavl, 150000 Russia; 6 All-Russian Research Institute of Medicinal and Aromatic Plants (VILAR), Grina St., 7, Moscow, 117216 Russia

**Keywords:** Oxidative stress, Inflammation, Zinc, Lipid metabolism, Glucose metabolism

## Abstract

A number of studies have reported that zinc plays a substantial role in the development of metabolic syndrome, taking part in the regulation of cytokine expression, suppressing inflammation, and is also required to activate antioxidant enzymes that scavenge reactive oxygen species, reducing oxidative stress. Zinc also plays a role in the correct functioning of lipid and glucose metabolism, regulating and forming the expression of insulin. In numerous studies, zinc supplementation has been found to improve blood pressure, glucose, and LDL cholesterol serum level. Deeper knowledge of zinc’s properties may help in treating metabolic syndrome, thus protecting against stroke and angina pectoris, and ultimately against death.

## Introduction

Zinc (Zn) is one of the most common trace elements in the human body and plays a substantial role in growth and development, acting as a signaling factor [1]. This metal takes part in the regulation of chronic inflammatory status through the reduction of inflammatory cytokines. Zinc also reduces oxidative stress by participating in the synthesis of antioxidant enzymes and acts as a catalyzer of enzymes, taking part in lipid, carbohydrate, and protein metabolism. It is involved in the synthesis, storage, and release of insulin, which suggests the critical role of this microelement in the progression of type-2 diabetes mellitus, atherosclerosis, and metabolic syndrome (MS) [2–5].

Studies of zinc concentration in the human body are scarce, and have shown inconsistent results. Zinc deficiency has been observed in patients in some counties with increased oxidative stress and generation of inflammatory status and the decrease concentrations of this element occur in patients with MS [3], in patients with type 2 diabetes mellitus [6] and with hypertension [7] than in healthy people.

Some studies showed that high concentration of zinc is associated with impaired lipid profile and risk of MS [8–10]. In the last study, increase in erythrocyte zinc concentration and high zincuria was observed in patients with MS [11]. Obtained results indicate that zinc is strongly associated with oxidation stress, inflammation, and lipid and glucose status and it can be assumed that zinc status may be a predictor of metabolic disorders.

Taking together, current knowledge reflects the need for a critical overview of the zinc role in metabolic disorders. The proposed review summarizes the advances of the last years (2010–2017), providing new insights into the association between zinc status and inflammation, oxidative stress, lipid, and glucose metabolism.

### Zinc status in metabolic disorders

Multiple studies have demonstrated the interaction between obesity and Zn homeostasis. In particular, blood Zn levels were found to be significantly decreased in obese patients [12, 13]. Erythrocyte Zn levels were also shown to be negatively associated with anthropometric markers of obesity like BMI and waist circumference [14]. At the same time, the decrease in serum Zn levels was accompanied by increased urinary concentrations, being indicative of increased Zn excretion in obesity [15]. Low nutritional Zn status in obesity is also associated with aggravation of obesity-related metabolic disturbances like insulin resistance, inflammation, and altered lipid profile [16].

Similarly, short-term (8-week) weight loss in obese women was associated with a significant improvement of serum Zn levels, being negatively associated with body fat percentage [17].

The role of Zn dyshomeostasis in obesity is also confirmed by the results of supplementation trials. In particular, administration of 30 mg/day Zn gluconate for 1 month resulted in a significant decrease in body weight and BMI values as well as serum TG concentrations [18]. Eight-week treatment with 20 mg/day zinc also resulted in a significant decrease in BMI and BMI *z* score in obese children, although remaining abnormally high. At the same time, Zn supplementation was also associated with improvement of lipoprotein profile (decreased ApoB/ApoA1, oxLDL, total cholesterol and LDL-cholesterol values) and reduced leptin levels [19].

It is also suggested that zinc deficiency may be an important risk factor of diabetes mellitus II. In several studies, decreased concentration of zinc status was observed in diabetic patients compared to healthy people [20–26]. Sinha et al. [20] have demonstrated that zinc plasma levels are inversely correlated with glycemic status (HbA1C) in diabetes mellitus, while in patients with MS an association between high zinc concentration in urine (zincuria) and fasting glucose level, glycated hemoglobin level, insulin resistance, and also CRP were found. In one study, inadequate zinc intake was observed and zinc deficiency was suggested in patients with MS [27]. The relation between inadequate zinc intake and raised insulin concentration in blood was also noticed in adolescents [28]. Some recent studies showed that zinc supplementation improved glucose metabolism and insulin sensitivity in diabetic patients [29–31]. It was also found that zinc supplementation reduced fasting plasma glucose, serum insulin and insulin resistance in gestational diabetes in women [32]. However, in other studies, the association between zinc supply and glucose metabolism and insulin resistance was not confirmed [18, 33].

In our opinion, the association between metabolic disorders and zinc status is mainly mediated by inflammation, oxidative stress, modulation of zinc transporters, and altered lipid and glucose metabolism.

### Inflammation

**Fig. 1:**
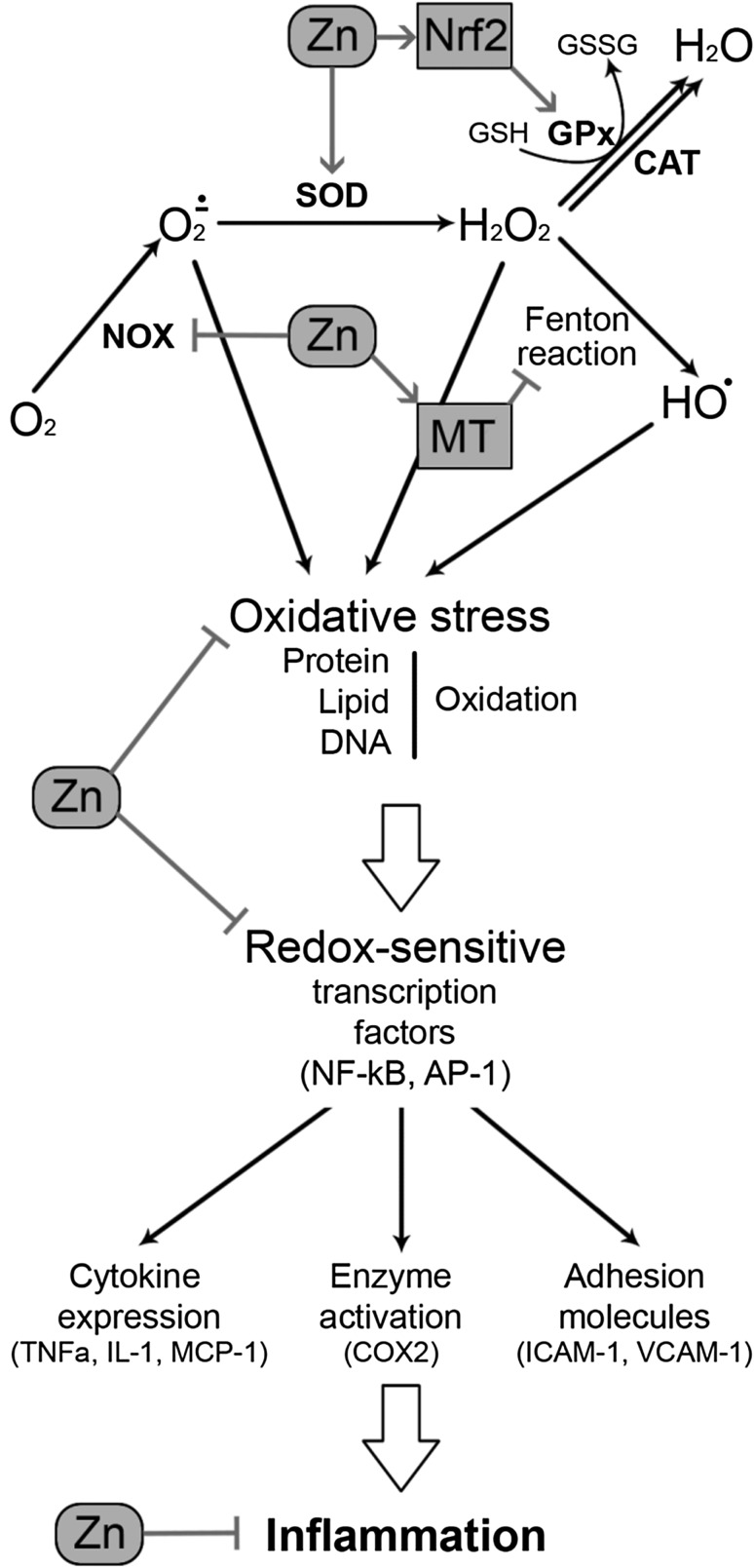
The influence of zinc on oxidative stress and inflammation. Zinc prevents the generation of reactive oxygen species through a number of mechanisms. Zn is a cofactor of Cu,Zn-SOD, which catalyzes the dismutation of superoxide. It up-regulates Nrf2, being the principal regulator of antioxidant system functioning, thus increasing GSH synthesis, GPx activity, and other mechanisms of detoxification. Zinc also affects the generation of reactive oxygen species through modulation of prooxidant pathways. Zn-induced decrease in NADPH oxidase activity results in decreased superoxide production. Through induction of metallothionein synthesis, Zn may decrease the availability of redox metals and their participation in Fenton reaction. Susceptibility of proteins to free radical oxidation is also inhibited by zinc through protection of thiol groups. Zn-induced inhibition of redox-sensitive proinflammatory transcription factors is accompanied by decreased production of proinflammatory cytokines, enzymes, and adhesion molecules, thus resulting in the prevention of inflammatory response

A significant decrease in zinc levels in obese population with inflammatory state was observed. According to research, it increases risk of the development of obesity-related complications. It has been mentioned that zinc takes part in the regulation of proinflammatory cytokines expression through many mechanisms (Fig. 1) [34].

During inflammatory state, white adipose tissue produces cytokines such as (interleukin-6) IL-6, which stimulates the secretion of C-reactive protein (CRP) in the liver, which is a sensitive marker of inflammation, tissue damage, and impairs vascular endothelial function. On the other hand, TNF-α contributes to the acute-phase response by enabling IL-6, which increases proinflammatory cytokines such as adiponectin [35]. In a double-blinded, placebo trial in elderly subjects, Bao et al. [27] have shown a significant increase in plasma zinc levels, a decrease in CRP, TNF-α, and Il-6 plasma concentrations in a response of the zinc supplementation. While different studies have shown the inverse association between IL-6, TNF-α, CRP, and serum zinc levels in adults aged 40 [36]. According to the authors, zinc supplementation positively impacts inflammatory state reduction in obese patients with metabolic syndrome. Also, in type 2 diabetes mellitus patients, impaired zinc homeostasis leads to uncontrolled expression of immune mediators, such as IL-1β, IL-6, and NF-κB, which at the same time simultaneously exacerbate the immune response and lead to pathogenesis, negatively affecting β-cells. The long-term exposure of β-cells to IL-1β and IL-6 may lead to apoptosis, resulting in insulin resistance and increased glucose levels in the blood [37].

NF-κB is one of the major immune response transcription factors in the development of atherosclerosis [35]. This inflammatory pathway takes part in the expression of pro-inflammatory cytokines, CRP, MMPs, and controls genes, which regulate proliferation, apoptosis, cell adhesion, tissue remodeling, inflammatory processes, immune responses, and cellular-stress responses [38]. Some authors have shown that zinc ions lead to signal transduction, thus making zinc involved in NF-κB inhibition [34]. Another negative impact of zinc regarding NF-κB activity appears through inhibition of lipopolysaccharide-induced activation of NF-κB, which in the same time suppresses TNF-α secretion in monocytes [39]. Also, zinc ions imported to macrophages or monocytes by ZIP8 (zinc transporter) during inflammatory state provokes NF-κB inhibition [40]. It seems that ZIP8 plays a crucial role in inflammation.

The main negative regulator of NF-κB activity is zinc finger protein (A20), which is known as a cell protector against TNF-α-induced NF-κB toxicity, decreasing its level, along with IL-1β. Prasad et al. [41] have demonstrated that this zinc–protein complex suppress TNF-α and IL-1β production, inhibiting the activity of NF-κB in endothelial cells. It has been reported that zinc supplementation reduces the level of these cytokines, reactive oxygen species (ROS), and polysaccharides by increasing the concentration of A20 and PPAR-α. Zinc influences the expression of peroxisome proliferator-activated receptors α (PPARs-α), which plays a significant role in lipoprotein and glucose metabolism. These receptors also suppress NF-κB activity. The inhibition of NF-κB by zinc via A20 and PPARs signaling pathways are the most important mechanism because zinc decreases pro-inflammatory cytokines in atherosclerosis [35].

Zinc also plays a key role in regulating the function of MMP2 and MMP9 named gelatinases whose expression increases during inflammation with artery damage. They belong to the group of zinc-dependent matrix metalloproteinases (MMPs), which bind zinc ions to the catalytic site for their activation, forming coenzymes. MMPs cause the degradation of various components of ECM and they mediate its remodeling, which enables cell migration, and facilitates the pathogenesis processes. Thus, they play an important role in immunity and angiogenesis, whereby their dysregulation may contribute to inflammation or atherosclerosis [42, 43]. Jagadeesha et al. [44] have reported that the activation of zinc-dependent endopeptidase MMP9 enables cell migration, while other authors have shown significant increasing of the MMP9 serum levels and lower zinc concentration in patients with unstable atherosclerotic plaque [45]. According to research, high MMP9 serum concentrations may increase the risk of atherosclerosis. Instead, some studies have revealed that zinc supplementation with a high-fat diet inhibits MMP2 and MMP9, decreases the inflammatory state, and lowers TG, LDL, IL-6, and CRP and increases HDL, as well as protecting the liver in rabbits [46]. Obtained results highlight the protective role of zinc against artery damage and atherosclerosis.

### Oxidative stress

Oxidative stress plays a significant role in MS development, being tightly associated with inflammation, and providing a link between certain MS components like obesity, diabetes, dyslipidemia, and hypertension. Moreover, recent studies proposed that oxidative stress may be considered as one of the components of MS [47].

Physiological concentration of zinc inhibits the production of reactive oxygen species, such as superoxide anion (·O−), hydrogen peroxide (H_2_O_2_), and radical hydroxyl (OH·) [48] as well as reactive nitrogen species including peroxynitrite (Fig. 1) [49]. The antioxidant effect of zinc may be mediated through direct action of zinc ion, its structural role in antioxidant proteins, and modulation metallothionein induction. Direct antioxidant activity of Zn ions is associated with its binding to thiol groups, thus protecting them from oxidation [50, 51].

Zinc is a cofactor of antioxidant enzyme Cu,Zn-superoxide dismutase (SOD1), which is suppressed under Zn-deficient conditions [52]. It has also been demonstrated that Zn may indirectly affect the activity of other antioxidant enzymes. In particular, Zn supplementation significantly increased GPx activity through modulation of Se status [53]. It has also been demonstrated that nanoformulated Cu,Zn-SOD is capable of decreasing adipose tissue [54] and vascular [55] inflammation in obese mice.

According to Manea et al. [56], the inhibition of NF-κB, in whose regulation zinc plays a critical role, holds up the expression of NOX1 and NOX4, which play an important role in redox status determination in blood vessels. They cause damage to vessels through by increasing the number of thrombins, causing NOX activation and elevated production of ROS, which leads to DNA and lipid oxidation, concurring to atherosclerosis [57], which has also been confirmed in Jagadeesha et al.’s [44] research. It is also notable that free zinc deficiency contributes to the inhibition of the *N*-methyl-d-aspartate receptor (NMDAR), which leads to an increased level of ROS [35, 58–60].

As stated earlier, Zn-dependent modulation of the antioxidant system is at least partially mediated by its influence on transcription factors. Nuclear factor erythroid 2-related factor 2 (Nrf2) is one of the key transcription factors regulating antioxidant system activity. It protects against oxidative stress at an early stage, scavenging reactive oxygen and nitrogen species, while zinc regulates its expression and transcription [58]. Li et al. [58] have demonstrated that zinc deficiency suppresses Nrf2 activity in diabetes-induced renal oxidative damage in mice. The intracellular concentration of zinc is partly regulated through MTs and closely linking the redox status of the cell to cellular availability of zinc ions [61].

It has been demonstrated that obesity-associated induction of MT expression in adipose tissue is a protective response, being at least partially mediated through modulation of oxidative and endoplasmic reticulum stress [62]. However, another group of authors has linked increased adipose tissue MT expression with the rate of insulin resistance [61]. Corresponding to the earlier-discussed role of Zn in MT synthesis, Zn intake and Zn status in obese patients were significantly associated with peripheral blood mononuclear cell MT levels [16]. Experimental studies have also demonstrated the impact of Zn status on antioxidant systems in obesity. In particular, Chen et al. [63] have assessed the effect of zinc deficiency and zinc supplementation on antioxidant expression in high-fat diet mice, inducing vascular inflammation and oxidative stress, demonstrating that zinc insufficiency exacerbates antioxidant expression, whereas zinc supplementation improves that expression.

It is worth mentioning that zinc does not always act as an antioxidant. In terms of high intracellular zinc levels, it can possess prooxidant properties. In particular, it has been demonstrated that zinc oxide nanoparticles significantly increased oxidative stress in 3T3-L1 adipocytes in a dose-dependent manner [64], although increasing the expression of antioxidant enzymes [65].

Some studies have demonstrated a tight interaction between metabolic syndrome and its components and the activity of Zn-dependent antioxidant systems. In particular, low serum SOD activity and zinc intake were shown to be associated with an incidence of MS in Shanghai, China [66].

Taheri et al. [67] reported decreasing activities of SOD and increases in GSH-Px (glutathione peroxide) and GR activities in diabetic patients. Correspondingly, serum Zn and SOD levels are significantly inversely correlated with HbA1c levels [68]. Hayens et al. [61] and Ogawa et al. [48] have demonstrated an elevated concentration of metallothionein 2a gene expression in diabetes individuals [61] and rats [48]. In contrast, Bellomo et al. [60] reported decreases in MT mRNA levels in response to high glucose concentration in β-cells in a mice model. Numerous experimental studies have demonstrated that Zn supplementation causes increased MT expression [69, 70]. However, it has been noted that in type 2 diabetics with normal Zn status, supplementation did not improve oxidative status and glucose metabolism [71].

Cu,Zn-SOD was also shown to play a significant role in altered lipid profile in diabetic patients [72]. Decreased activity of Cu,Zn-SOD in relation to Zn-deficiency was observed in hypertensive patients. Certain studies have demonstrated increased SOD activities in metabolic syndrome and its components. In particular, Vávrova et al. [73] have shown altered antioxidant enzymes levels in patients with metabolic syndrome, including significantly increased Cu,Zn-SOD and glutathione reductase (GR) activities, and lower activities of CAT and PON1 (paraoxonase) in association with low GSH levels. Similarly, erythrocyte SOD activity was increased in type 2 diabetics, being significantly associated with Zn levels and glycemic control. The authors state that the increased enzyme activity is a response to counteract oxidative stress in terms of adequate zinc supply [74].

Therefore, zinc has a protective potential against metabolic syndrome-associated oxidative stress through induction of transcription factors (including (ARE)-Nrf2 signaling) and a subsequent up-regulation of enzymatic and non-enzymatic antioxidants, induction of metallothionein synthesis, its structural role in Cu,Zn-SOD (SOD1), and, finally, its direct antioxidant activity. It seems that metabolic stress during obesity and metabolic syndrome induces a compensatory response, being characterized by increased Zn-mediated mechanisms of antioxidant protection. However, in terms of poor Zn status due to increased Zn excretion or insufficient intake, their mechanisms may not be activated, resulting in aggravation of metabolic disturbances.

### Lipid metabolism

Adipose tissue is the main depot of the lipids in the human organism and expanded adipose tissue mass due to overaccumulation of lipids is the morphological substrate of obesity. Therefore, a tight interaction between Zn and adipose tissue dysfunction is of particular interest. Numerous studies have indicated an association between serum zinc levels and lipid metabolism [75]. In clinical and experimental studies, it has been reported that zinc supplementation results in the total cholesterol, LDL cholesterol, and triglycerides decreasing, and the HDL cholesterol increasing in patients [29, 52]. Instead, Weigand and Egenolf [76] have shown that moderate zinc deficiency did not alter lipid concentration and fatty acid composition in the liver of rats fed a high-fat diet. In other studies, zinc deficiency exacerbates hepatic lipid metabolism, while Zn supplementation increases hepatocyte activity and improves lipid metabolism in the liver [77, 78]. Moreover, short-term zinc supplementation in obese patients decreases weight and TG levels without significant change in lipid and glucose profile [18, 79, 80].

Numerous studies have demonstrated that the state of adipose tissue in obesity and other pathologies is tightly associated with Zn status. Experimental studies have demonstrated a significant decrease in adipose tissue Zn content and its negative correlation with insulin, HOMA-IR, and TNF-α values in obese animals [81]. A significant decrease in adipose tissue Zn levels was detected in animals fed both Zn-adequate and Zn-deficient high-fat diets. At the same time, dietary Zn deficiency in overfed animals resulted in a significant increase in serum leptin levels, being accompanied by a more intensive macrophage infiltration as compared to the HFD-Zn-adequate group [82]. In opposite, other studies showed that a high-fat diet significantly elevated the zinc level in plasma in rats [83].

It seems that Zn does not passively react to the changes in adipose tissue mass. Although being rather contradictory, the existing data demonstrate that the metabolic effects of Zn in obesity may be associated with its interference with leptin production.

Experimental studies demonstrate that adequate adipose tissue Zn status is required for normal adipocyte functioning and leptin synthesis to provide a leptin-mediated negative feedback. Taking into account the fact that Zn supplementation was associated with a further increase of serum leptin levels in obese leptin-resistant subjects, being accompanied by improvement of weight and metabolic parameters, one can propose that Zn may also decrease the rate of leptin resistance. At the same time, Baltaci and Mogulkoc have proposed that leptin may act as a possible link between zinc and immunity [84].

Zinc has a significant impact on other adipokines. In particular, it has been demonstrated that Zn stimulates oligomerization of higher molecular weight forms of adiponectin through modulation of disulfide bond formation [85]. These findings are in agreement with the clinical observation of a positive correlation between serum Zn and adiponectin levels in obese patients with polycystic ovary syndrome [86]. Moreover, 50 mg/day of Zn supplementation for 12 weeks in obese examinees was associated with a significant more than twofold increase in serum adiponectin concentration [87].

**Fig. 2:**
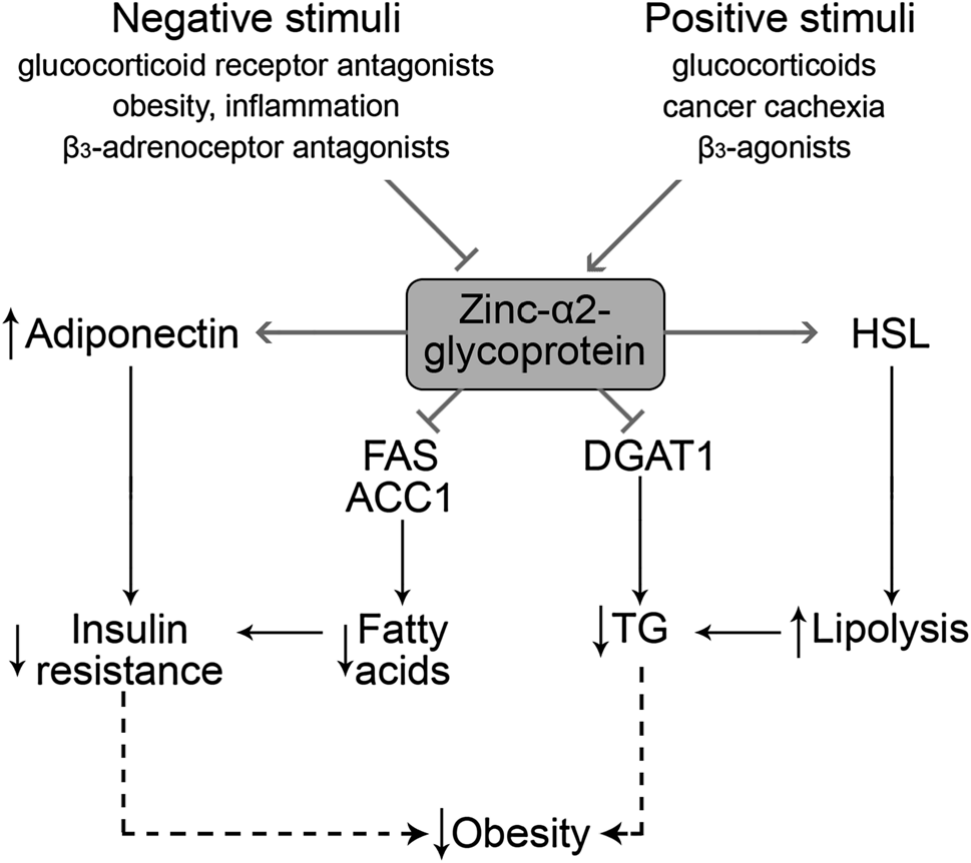
Functions of ZAG in adipose tissue. Expression of ZAG is down- or up-regulated by the negative or positive stimuli. In adipose tissue, ZAG inhibits FAS and ACC1 activity, thus resulting in decreased fatty acid synthesis. Lower level of free fatty acids together with ZAG-induced increase in adiponectin expression may significantly reduce insulin resistance. Depression of DGAT1 activity suppresses triglyceride synthesis. Together with up-regulation of HSL the latter results in lower lipid deposition in adipose tissue

Zinc-α 2-glycoprotein (ZAG), a novel adipokine, was shown to be inhibited by a high-fat diet and obesity, inflammatory stimuli (TNF α), glucocorticoid receptor antagonists, eicosapentaenoic acid, and β3-adrenoceptor antagonists, whereas glucocorticoids, cancer cachexia, and β3-agonists increase ZAG production [88]. The physiological effect of ZAG in adipose tissue is related to regulation of lipid metabolism. In particular, ZAG decreases fatty acid synthase (FAS), acetyl-coenzyme A carboxylase 1 (ACC1), acyl-coenzyme A: diacylglycerol transferase 1 (DGAT1), and increases hormone-sensitive lipase (HSL) activity, resulting in increased lipolysis and decreased lipogenesis in murine adipose tissue [89]. Antiobesity effect was confirmed by Russell et al. [90], who reported that decreasing the concentration of ZAG induces lipolysis, which manifests as increasing glycerol levels in rat plasma. They have also demonstrated elevated lipid utilization as a result of decreasing TG levels [90, 91]. According to Yang et al. [92], ZAG is also correlated with insulin resistance. These authors have found lower levels of this adipokine in obese patients with type 2 diabetes mellitus. Inverse associations between insulin resistance, obesity, leptin mRNA, and ZAG mRNA levels have also been found by Mracek et al. [93], who assumed that ZAG has a protective affect against obesity, and as a consequence, against metabolic syndrome, too. Mracek et al. [94] have also revealed that TNFα exhibits a repressive effect on ZAG expression in differentiated adipocytes, which suggests that inflammation exacerbates ZAG operation. Zhu et al. [95] have confirmed that, in hypertensive patients, the ZAG level decreases and the TNFα level increases at the same time. Russell et al. [90] have documented lipolytic activity of ZAG and reductions in body fat mass during treatment of mice for 15 days with ZAG. The effects of ZAG on adipose tissue metabolism may also be mediated through a positive influence on adiponectin expression [96]. Taking into account the presence of zinc-binding sites on zinc-α 2-glycoprotein molecule and its role in adipose tissue physiology [97], one can propose that ZAG may mediate at least a part of the effects of Zn in obesity. Figure 2 shows the schematic diagram of ZAG function in adipose tissues.

The targets for Zn-mediated impact on energy metabolism may include PPARs (proliferator-activated receptors), which are responsible for the expression of mRNA genes that play a critical role in energy metabolism [98, 99]. Three types of PPAR can be singled out: PPARα, PPARβ/δ, and PPARγ. PPARα and PPARβ/δ are mainly responsible for regulation of fatty acid degradation, whereas PPARγ takes part in lipid storage, regulates adipocyte differentiation, and enhances adipogenesis and insulin sensitivity [100–104]. The significant role of zinc in the zinc-finger protein as part of the proper functioning of PPARs has been confirmed by the research of Zhou et al. [101].

The observed interactions between Zn status and adipokine production are dependent on the functional state of the cell. In particular, certain studies have indicated that modulation of Zn levels has a significant impact on adipose tissue differentiation. A recent study demonstrated that zinc oxide (ZnO)-treatment (1–4 mg/l) upregulated PPARγ, FABP4, C/EBPα, and SREBP1 mRNA expression and stimulated lipid accumulation in adipocytes during adipogenesis [105]. Similar findings were obtained for zinc-chelated vitamin C [106]. In contrast, Justus et al. [107] have shown that zinc deficiency does not exacerbate PPARγ gene expression in rats. At the same time, certain studies demonstrated that ZnO nanoparticles (> 10 µg/ml) may be toxic to adipose tissue-derived mesenchymal stem cells, decreasing cell viability through induction of apoptosis [108].

The impact of Zn on adipocyte differentiation may be related to the functioning of various Zn-containing proteins that have been recognized as early regulators of adipogenesis [109]. In particular, it has been demonstrated that Zn-finger protein ZNF638 is induced at early stages of adipocyte differentiation and stimulates adipogenesis through C/EBPs and subsequent up-regulation of PPARy. In turn, ZNF638 knockdown inhibits adipogenesis [110]. Another Zn-finger protein, Zfp423, was also shown to be the factor stimulating adipocyte differentiation acting via stimulation of PPARy expression. It is also notable that Zfp423 deficiency is associated with impairment of both white and brown adipose tissue development [111]. Zfp467 stimulates progenitor cell differentiation from osteoblastic to adipocyte lineage, increasing PPARy, C/EBPa, adiponectin and resistin expression [112]. At the same time, Zfp467 suppression stimulated osteoblast commitment and alleviated osteoporosis, being characterized by specific changes of adipogenic and osteogenic marker proteins [113]. In turn, Zfp521 acts as a negative regulator of adipogenesis at least partially through inhibition of Zfp423 expression [114]. Other Zn-finger proteins involved in regulation of adipocyte determination and differentiation include Znf395, Shn-2, GATA proteins, SLUG, Egr2/Egr1, ZBTB16, YY1, and Krüppel-like factors [115].

### Zinc and insulin secretion

Zinc is an essential trace element required for the normal synthesis, storage and secretion of insulin in pancreatic β-cells. It has been reported in a recent study that the depletion of zinc negatively impacts insulin sensitivity and glucose tolerance [116]. Instead, Jayawardena et al. [117] have shown that zinc supplementation improves glucose homeostasis in patients with diabetes. Ahn et al. [118] have reported that zinc concentrations are inversely associated with insulin resistance but not correlated with metabolic syndrome. This metal also stimulates glycolysis, inhibits gluconeogenesis, and plays a role in glucose transport in adipocytes [119].

Insulin is co-stored and co-crystallized in granules in pancreatic β-cells with free cytosolic zinc. Six insulin monomers congregate in β-cells into a hexamer with two zinc ions in the center, and this form is stored and transported across the cell membrane as an insulin-zinc crystal in the normal functioning of β-cells. Slepchenko et al. [120] have shown inhibition of the zinc feature upon further glucose-stimulated secretion of insulin. After the zinc–insulin complexes have been secreted, they start to dissociate; only the insulin monomer is an active form of the hormone. The elevated extracellular concentration of free zinc is related to the increase in insulin secretion [60, 120–122].

Along with insulin, zinc also takes part in the inhibition of glucagon secretion in response to high glucose concentrations. Glucagon is a hormone secreted by α-cells of the pancreas, which increase the glucose blood level during hypoglycemia. When the glucose concentration decreases, zinc is released from the β-cells with insulin, triggering glucagon secretion [121]. These findings were confirmed in the research conducted by Slucca et al. [123] and Myers [124]. These authors have confirmed the inhibitive properties of zinc released with insulin on glucagon function in mice. Glucagon also regulates glycogen breakdown and gluconeogenesis and decreases at the same time triglyceride synthesis by the liver [11].

### Zn transporters

The interaction between zinc status and obesity may be at least partially mediated by obesity-induced modulation of zinc transporters that regulate cellular and intracellular Zn fluxes [1]. In particular, in obese women, the highest expression was observed for ZnT1 followed by Zip1, whereas no significant expression of Zip3 was found [125]. It has been revealed that obese patients were characterized by a significantly lower expression of Zip14 in subcutaneous adipose tissue, whereas a 10-week weight loss period significantly increased gene expression. Zip14 expression directly correlated with PPARy expression and HDL-C concentration, although being negatively associated with anthropometric markers of obesity, body fat percentage, HOMA-IR, blood glucose, insulin, and TG levels [126].

It was also observed that the deficiency of zinc-transporter protein 7 (ZnT7) plays a substantial role in lipid metabolism [127]. It was found that the ZnT7 expression was induced by lipogenic differentiation in ZnT7 knockout mice and the decreased expression of this transporter exacerbates the signal transduction pathway activity, which regulates basal and insulin-stimulated glucose uptake in adipocytes [128].

**Fig. 3:**
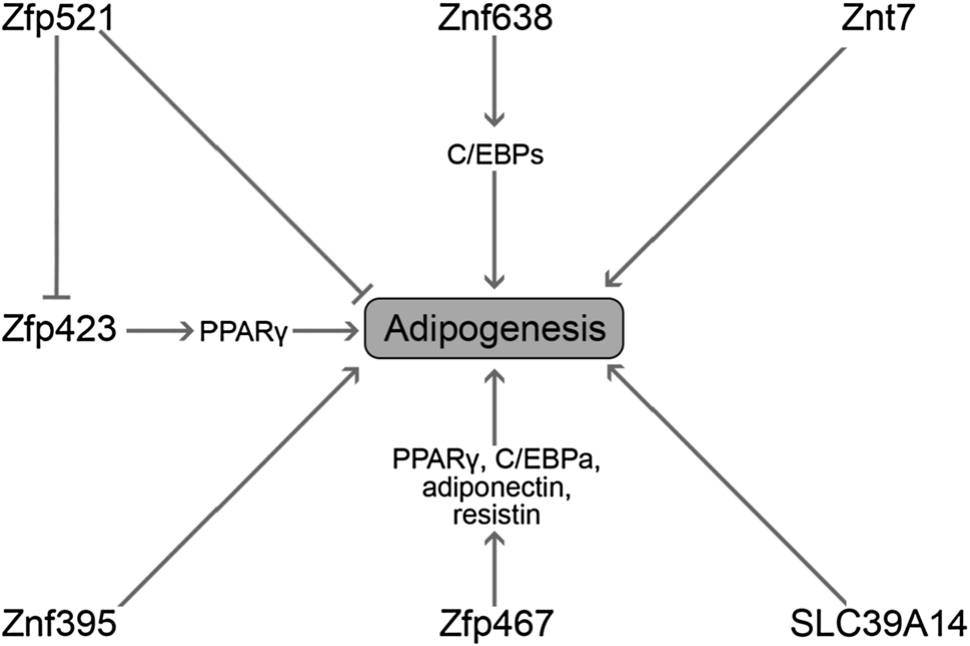
The role of zinc finger proteins and zinc transporters in the regulation of adipogenesis

It has also been demonstrated that SLC39A14, a member of the ZIP protein subfamily, is induced during the early stages of adipogenesis, being associated with increased Zn uptake [126]. It is also notable that Zip14 regulates inflammatory signaling in adipocyte hypertrophy [129]. These findings are in agreement with the earlier data indicating a transient increase of intracellular Zn levels in 3T3L1 cells at the transition from G0/G1- to S-phase of the cell cycle, followed by a decline to the initial levels. Moreover, significantly lower ZnT4, ZnT5, ZnT9, Zip1, Zip4, and Zip6 mRNA levels in lymphocytes were revealed in obese Korean women. Moreover, ZnT4, Zip1, and Zip6 mRNA levels were inversely associated with CRP concentration, whereas ZnT4 and ZnT5 mRNA levels were characterized by an inverse correlation with TNF-α [130]. It has recently been shown that ZIP13 is a crucial regulator of beige adipocyte biogenesis and thermogenesis. Inhibition of ZIP13 function enhanced beige adipocyte biogenesis and energy expenditure by regulating C/EBP-β expression in mouse [131]. It seems that the association between zinc homeostasis and brown adipose tissue, thermogenesis, and inflammation may contribute to the development of new therapies for obesity and metabolic syndrome. Figure 3 summarizes the role of zinc-finger proteins and zinc transporters in regulation of adipogenesis.

It is suggested that also in glycemic control and glucose homeostasis transport zinc proteins play a crucial role. ZnT8 seems to be one of the most important zinc transporters in diabetes. Zinc-protein (ZnT8) is mainly expressed in pancreatic INS-1 β-cells, which contain the largest amount of zinc; this complex induces glucose-stimulated insulin secretion. ZnT8 plays an important role in zinc transport from the cytoplasm into insulin secretory granules within islets, and is thus an essential transporter in the synthesis, storage, and function of insulin [124, 132, 133]. Wijesekara et al. [134] and Pound et al. [133] have shown that zinc deficiency in mice may lead to ZnT8 depletion and may contribute to perturbed function of the islets of Langerhans and increased of insulin, leading to a greater risk of type 2 diabetes mellitus. These authors also reported that ZnT8 depletion may trigger the formation of atypical insulin-zinc secretory granules [133, 134]. Furthermore, Merriman et al. [135] have found that even mutations in ZnT8 may cause greater risk of type 2 diabetes.

Zinc also plays a crucial role in insulin degradation in the liver. It has been found that mutation in ZnT8 may contribute to dysregulation in insulin in the first passage through the liver and increase risk of type 2 diabetes mellitus development [136]. Huang et al. [137, 138] demonstrated that ZnT7 transporter is also implicated in glucose–insulin homeostasis.

Βeta-cells contain larger amounts of zinc ions than are required for the formation of the zinc–insulin granules. After the co-crystallization and secretion of the insulin–zinc granules, the remaining quantities of zinc are redistributed to the cytosol by Zip transporters [139, 140]. Hardy et al. [139] have demonstrated increased levels of free cytosolic zinc in response to a higher level of Zip-4 in mice, whereas Liu et al. [140] reported an elevated concentration of zinc ions in response to increased activity of Zip-6 and Zip-7 transporters. Myers et al. [141] attempted to find a connection between Zip-7 activity and carbohydrate homeostasis; they demonstrated the contribution of Zip-7 to glucose uptake and the storage of glycogen in skeletal muscles. They also conclude that this transporter may be used in insulin resistance treatment. Feitosa et al. [142] have shown that Zip-14 also takes part in zinc homeostasis during inflammation caused by obesity. They also reported elevated plasma concentrations of IL-6, leading to the increased expression of Zip-14 in obese women.

Recent studies have brought attention to the fact that zinc acts as a signaling factor. The mechanism of zinc’s insulin-mimetic activity has been observed in several studies on glucose and lipid metabolism [124]. The signal–transduction mechanism of ZIP10 reflects the role of Zn signaling in B cell function. Zip10-KO mice study showed evidence that ZIP10 signaling regulates caspase activity, promotes the survival of pro-B cells, and regulates the function of mature B-cells [1].

## Conclusions and perspectives

Zinc is an essential trace element that plays a substantial role in the prevention of metabolic syndrome, including atherogenic dyslipidemia, hyperglycemia, insulinemia, and elevated blood pressure through the inhibition of proinflammatory cytokine expression, which suppresses ROS production, protecting against oxidative stress damage. Zinc takes part in ROS neutralization as well as in glucose and lipid metabolism. Zinc is thus highly significant in the pathogenesis of metabolic syndrome, which suggests that zinc supplementation would have a positive effect in regressing metabolic syndrome.

Further investigations of the relationship between Zn and cytokines, adipokines, antioxidants, and receptors are needed to explain the role of zinc in health and diseases. Modulation of zinc status may become a new target in the prevention and treatment of metabolic disorders. It has been shown that Zn transporters play the crucial role in zinc homeostasis in the body [1]. It seems that deeper knowledge about physiological functions of Zn transporters and the ability to control their activity may be an important factor in developing new therapies for Zn-related diseases.
